# Spatial Ecology of an Arboreal Iguana (*Oplurus cyclurus*) in a Treeless Landscape

**DOI:** 10.3390/ani13203198

**Published:** 2023-10-13

**Authors:** Fulvio Licata, Paolo Eusebio Bergò, Devin Edmonds, Franco Andreone, Gonçalo M. Rosa

**Affiliations:** 1 Centro de Investigação em Biodiversidade e Recursos Genéticos (CIBIO), InBIO Laboratório Associado, Universidade do Porto, Campus de Vairão, 4485-661 Vairão, Portugal; 2 Biopolis Program in Genomics, Biodiversity and Land Planning, CIBIO, Campus de Vairão, 4485-661 Vairão, Portugal; 3 Museo Regionale di Scienze Naturali, 10123 Torino, Italyfranco.andreone@gmail.com (F.A.); 4 Illinois Natural History Survey, Prairie Research Institute, Champaign, IL 61820, USA; 5 Department of Natural Resources and Environmental Sciences, University of Illinois Urbana-Champaign, Champaign, IL 61801, USA; 6 Institute of Zoology, Zoological Society of London, London NW1 4RY, UK; 7 Centre for Ecology, Evolution and Environmental Changes (cE3c) & Global Change and Sustainability Institute (CHANGE), Faculdade de Ciências da Universidade de Lisboa, Campo Grande, 1749-016 Lisboa, Portugal

**Keywords:** Sauria, ecology, Isalo, Madagascar, radio-telemetry, home range, site fidelity

## Abstract

**Simple Summary:**

The spiny-tailed lizard *Oplurus cyclurus* is a widespread endemic iguanian occurring in southern and western Madagascar dry areas. This species is mostly arboreal, and little is known about its spatial ecology. We conducted a radio tracking study on 19 individuals of a population with saxicolous habits, inhabiting an open, treeless savannah in the Isalo sandstone massif (central-southern Madagascar). Tracked lizards had a small home range size (95% isopleth = 247.8 m^2^) and showed high site and burrow fidelity. The activity pattern was unimodal, increasing along the day and with juveniles more active than adults in unfavourable weather conditions. Basking occurred mostly near the burrow entrance. Despite high burrow fidelity, lizards changed shelters regularly (approx. once a week), but there was no obvious relation between lizards’ body and/or tail size and the width and depth of selected burrows. We argue that the saxicolous habits of this population may entail local behavioural adaptations.

**Abstract:**

Understanding the spatial ecology of species has important implications for conservation, as it helps identify suitable habitats and minimum requirements for biodiversity monitoring and management. The spiny-tailed lizard *Oplurus cyclurus* is a widespread endemic iguanid occurring in dry areas of southern and western Madagascar. While the species is known to be mostly arboreal, populations of the Isalo sandstone massif suggest local adaptation to a less forested savannah and a more exposed habitat. We radio-tracked 19 spiny-tailed lizards to investigate the species’ rock-dwelling behaviour and spatial ecology at Isalo National Park. Tracked individuals showed high site and burrow fidelity, and a basking behaviour mostly tied to the accessibility of their burrow, the time of day, and their life stage. Activity peaked during the sunniest hours, while juveniles were more active than adults with unfavourable weather conditions. Despite high burrow fidelity, lizards used shelters non-exclusively, regularly changing (approx. once a week) with neighbouring burrows (average distance between burrows = 13.6 m). However, there was no obvious relation between lizards’ body and/or tail size and the width and depth of selected burrows. Dynamic Brownian Bridge Movement Models estimated frequented areas over 247.8 m^2^ (95% isopleth), where territorial overlap is common. Our results challenge the notion that burrow-site fidelity is the sole driving factor behind space utilization in the studied population. We argue that the apparently unusual saxicolous habits imposed by habitat features (the absence of trees) may lead to local behavioural adjustments influencing antipredatory and foraging strategies, as well as intraspecific interactions.

## 1. Introduction

With the herpetofauna of Madagascar facing rapid ongoing habitat loss, the description of new reptile species is often a compulsory action so that species-based conservation measures can promptly be enacted. In particular, the IUCN Red List of Threatened Species requires species to be named to propose conservation categories and apply safeguard measures [1,2,3]. While much time and effort has been spent on taxonomy, outside a handful of popular species common in the pet trade (e.g., *Furcifer pardalis* [4]), the ethological and ecological requirements of most reptile species in Madagascar remain largely unknown. Indeed, compared to birds and mammals, reptile behaviour overall has been neglected by researchers [5], and for lizards, we are missing critical habitat use data for many species [6].

Iguanians are among the most diverse and species-rich groups of lizards, which occur in a vast range of habitats and have developed a plethora of life history strategies [7]. The family Opluridae contains eight species native to Madagascar, one of which also occurs in the Comoros [8]. Oplurid lizards are the only iguanians known outside the Americas and Pacific Islands [7]. Only a few studies have been conducted on *Oplurus* ecology and behaviour, with the majority concentrated on the better-known *O. cuvieri*. Research has revealed that this species primarily utilizes an ambush strategy to capture prey [9,10] and exhibits notable territorial behaviour and site fidelity [8], which is also observed in other *Oplurus* [11,12]. However, most advancements have been made within captive settings [13,14], leaving the understanding of *Oplurus* spatial ecology in the wild relatively unexplored. Oplurids include mostly saxicolous species (*O. grandidieri*, *O. fierinensis*, *O. saxicola*, and *O. quadrimaculatus*) and the arboreal sister species *O. cuvieri* and *O. cyclurus* [8]. The latter is known to have higher body activity temperatures than its sister species, making it more likely to inhabit warmer environments [15]. Furthermore, contrary to *O. cuvieri*, *O. cyclurus* is known to have at least in part terrestrial habits, as it sometimes catches its prey on the ground [15].

During fieldwork carried out at the Isalo Massif in central-southern Madagascar, we found a population of *O. cyclurus* with fully saxicolous habits. Here, instead of perching on tree branches, *Oplurus* lizards basked on piles of stones, large rocks, or close to burrows that were used as shelters in dry open grassland habitats, often near canyons. Investigating the spatial ecology of populations with different, unusual life habits may help to disentangle the role of habitat in determining behavioural, physiological, or life history changes. Furthermore, contextualising habitat features and the spatial ecology of species may also contribute to a better understanding of their ecological requirements. Thus, studies in these fields represent an exciting and much-needed area of research to advance our understanding of eco-evolutionary processes, providing useful insights into species’ ecological requirements for conservation purposes.

Saxicolous habitats are structurally simpler than arboreal ones, and may therefore impose different challenges to species by increasing exposure to predation, altering basking, foraging, and sheltering strategies, while affecting intraspecific interactions [6]. In this study, we hypothesized that saxicolous habits could drive a peculiar utilization of the space in the arboreal *O. cyclurus*. Using radiotracking, we investigated the spatial ecology of this species in an unusual habitat by focusing on (1) home range size, (2) activity patterns, and (3) burrow use.

## 2. Materials and Methods

### 2.1. Study Site and Period

Isalo is a large sandstone massif within the Ihorombe Region of central-southern Madagascar, ranging between 510–1268 m a.s.l. A network of canyons cuts through the stone matrix, in particular in the eastern and north-western sectors [16]. The climate of Isalo is sub-desertic and dry tropical, but some canyons are on the limit of the humid eastern and dry western biomes, hosting enclaves of humid forests. Around 850–1200 mm of rain falls every year, with 90% of the precipitation occurring between November and March [17]. A few rivers are permanent, whereas many others are seasonal. Temperatures vary greatly between monthly means: 17 °C in June and 25 °C in February [18].

The northernmost part of the massif is currently managed as a national park by MNP (Madagascar National Park) and is one of the largest protected areas of Madagascar, with 815 km^2^ of extension [19]. Extensive areas of the massif are covered by bare rock or savannahs, the latter being maintained by human actions through the centuries. Annual fires are set to manage the savannah to allow grazing for zebu cattle, which are bred in high numbers by the Bara ethnic group in this region [20]. Grass savannah is interspersed with a mosaic of tree savannah mostly represented by fire-resistant species (such as the palm *Bismarckia nobilis* and *Uapaca bojeri*), which sometimes form forest patches. In open areas, particularly on steep slopes or exposed ridges, vegetation is sporadic, dwarf and mainly xerophytic.

**Figure 1:**
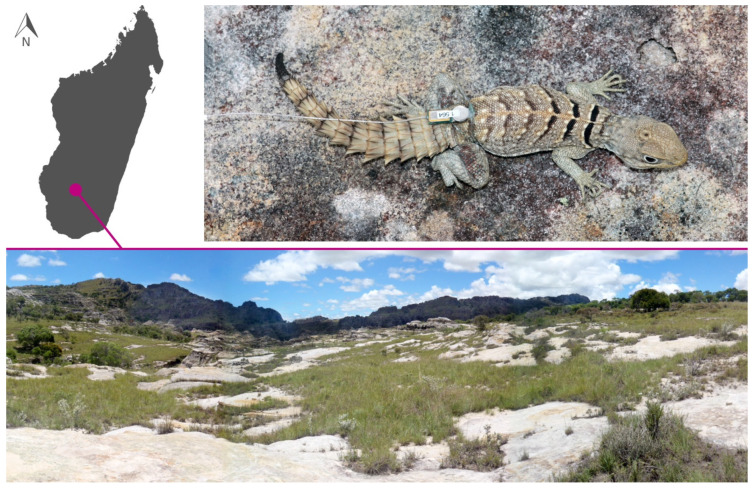
Study system: Study site (Malaso) in Isalo Massif, central-southern Madagascar (**bottom**); individual of *Oplurus cyclurus* with a radio-transmitter harnessed to its waist (**top**).

Data collection took place during a four-week period in January and February 2011, when increased rainfall tended to heighten foraging activity in Malagasy oplurids [9]. The study was conducted at a site locally known as Malaso (Figure 1; −22.59146°, 45.35744°; elevation 899 m), within the boundary of the national park. Malaso mostly consists of a wide rocky area surrounded by a largely treeless savannah and holds a narrow canyon (extending for about 260 m) below ground level, cut by a small stream. The internal parts of the canyon are deep, around five meters, and several small pools form along the stream bed. Canyons such as this assure a peculiar microclimate and micro-habitat that is consistently wetter and more humid than in the outside savannahs.

### 2.2. Radio-Tracking, Distance Measurements, and Environmental Variables

We used snares to capture lizards. Each individual was then weighed with an electronic scale (precision at 0.1 g) and their total length (TL) and tail length (TaL) was measured with a digital calliper to the nearest 0.1 mm. Once measured, we attached a radio-transmitter with a unique frequency harnessed to their waist. We used two kinds of transmitting devices: (1) the ATS A2414 glue-on-transmitter (Advanced Telemetry Systems, Inc., Isanti, MN, USA), and (2) the Holohil BD-2N transmitter (Holohil Systems Ltd., Ottawa, ON, Canada), both with a weight of approx. 0.3 g. We followed Tramontano [21] and affixed the transmitters externally with a cotton thread. The antenna was external, about ten mm long, and left trailing behind the hind legs (Figure 1). Individuals’ positions were obtained by homing-in [22], with a Biotrack SIKA receiver and a folding three-element Biotrack antenna.

Search and detection of the radio-tagged individuals was performed once a day. Signals ranged from a maximum distance of 75–120 m. Once located, we marked the position with a flag and recorded GPS coordinates. We then moved the flag and recorded new locations every session. We also recorded if a detected individual was “active” (basking) or “non-active” (hiding in the burrow), and then we measured the distance of the displacement of each individual in relation to the former location. Thus, we categorized locations as “displacement” if the lizards were found at a distance ≥ 50 mm from the former location. The distance between two consecutive contacts (despite the orientation) was measured linearly using a ribbon meter and in the subsequent analysis considered as a simple value (distance). We defined “basking distance” as the Euclidean distance of the basking location to the burrow frequented by the individual on the same day.

Cloud coverage was assessed daily and categorized into three levels: ‘clear’ (less than 30% sky coverage), ‘partially cloudy’ (30–70% sky coverage), and ‘cloudy’ (over 70% sky coverage). We obtained the hourly temperature and the wind speed at 1.2 m above ground level for the centroid of the Minimum Convex Polygons (MCP) including all lizard locations using the R packages *microclima* [23] and *NicheMapR* [24], which allow for producing realistic estimates of microclimate at fine (<30 m) spatial and temporal scales anywhere on Earth [25]. This method allows for obtaining terrain and sub-daily atmospheric forcing data from the National Centers for Environmental Prediction (NCEP) (using the R packages *elevatr* and *RNCEP* [26,27]), which are downscaled and interpolated through a microclimate modelling approach which account for terrain and shade adjustments [25].

### 2.3. Home Range Estimates

We used Dynamic Brownian Bridge Movement Models (dBBMMs) to calculate the home range of the lizards. This class of models provides occurrence distribution probabilities based on animal movement paths [28], accounting for temporal autocorrelation and incorporating errors associated with the radiotracking device used. Moreover, dBBMMs have been successfully used to estimate home range in reptiles with very different behaviours [29]. We set the window size and margin, which regulate the Brownian motion variance parameter, at 7 and 3 subsequent locations, respectively, to allow calculations for all individuals with more than 7 observations (*n* = 10). When time gaps between observations did not allow the computation of dBBMMs, we estimated the home range using MCP and Kernel Density Estimation (KDE) using the *href bandwidth* selection algorithm. To compute dBBMMs, we used the R package *move* [30], while for MCP and KDE we used the R package *adehabitatHR* [31].

### 2.4. Activity Pattern

We used binomial Generalized Linear Mixed Models (GLMMs) to test the relationship between activity status and time of observation (minutes from sunrise), including its quadratic term to assess non-linear relationships, the difference between adults and juveniles (i.e., TL more and less than 100 mm, respectively), and the effect of cloud coverage (no clouds, partially cloudy, and cloudy), temperature, and wind speed.

### 2.5. Burrow Use

We obtained the number of burrows used, the number of times individuals changed burrows, and the number of burrows revisited during the tracking period. To assess the temporal use of burrows, we assumed that consecutive observations in the same burrow did not include undetected displacements to other burrows, while single observations in a burrow were considered as a single day of burrow use. Furthermore, we measured the burrows’ width and depth and used Linear Mixed Models (LMMs) to test whether these burrows’ features (ln-transformed to reduce skewness) were related to TL and TaL of individuals.

### 2.6. Basking Behaviour

We used LMMs to test the relationship between the basking distance (ln-transformed to reduce skewness) and time of observation (minutes from sunrise), with its quadratic term, while taking into account the effect of the ontogenetic stages (adults vs. juveniles), cloud coverage (no clouds, partially cloudy, and cloudy), temperature, and wind speed.

### 2.7. Modelling Procedure

In all mixed models, we considered “individual” as a random effect to account for possible interindividual variation in the response variables. Prior to running the models, we assessed potential collinearity issues [32] between independent variables using (VIF) and its generalised version (i.e., gVIF [33]), which indicated a lack of multicollinearity issues (i.e., VIF and gVIF always <2). We built models using all combinations of explanatory variables and ranked them by corrected Akaike Information Criterion (AIC_c_) [34]. We used the ‘nesting rule’ to select the final set of candidate models, excluding all models which had a simpler nested model with lower AIC_c_ [35]. For each model, we calculated the AIC_c_ weight, which indicates the relative likelihood of a model given the data and the candidate set of models. Models were run in the *R* environment [36], using the package *lme4* for mixed effect models [37], and the package *MuMIn* for computing models with all combinations of explanatory variables (function ‘*dredge*’ [38]). For LMMs, test statistics were obtained using the *lmerTest* package [39], while for binomial GLMMs we calculated test statistics using the likelihood-ratio test (LRT) [34]. Data visualization was performed using *ggplot2* [40].

## 3. Results

We made 234 field observations on 19 *Oplurus* individuals, found over an area of 1.9 ha (100% MCP of all observations), which corresponds to 9.7 individuals per hectare. On average, we recorded 12 observations per individual (SD = 8; median = 8; range = 1–46; Table S1). The average time between observations was 21.6 h (SD = 16). Seventeen individuals were monitored for an average of 11 days each (SD = 7; median = 9; range = 1–30; see Table S1), while two were followed for less than 24 h (Table S1).

### 3.1. Home Range Estimates

The average home range size for nine individuals was 1036.6 m^2^ (SD = 1511.4; range = 36.0–4650.7) for the 99% dBBMM isopleths, 247.8 m^2^ (SD = 277.9; range = 11.7–762.1) for the 95% isopleths, and 40.9 m^2^ (SD = 48.8; range = 4–145.9) for the 75% isopleths (see Table S1). The size of the 99% isopleths (ln-transformed to reduce skewness) was correlated with the duration of the tracking period (Pearson’s correlation r = 0.8, df = 7, *p* = 0.002), but it was not correlated with the body size of the individuals (Pearson’s correlation r = 0.3, df = 7, *p =* 0.491).

**Figure 2:**
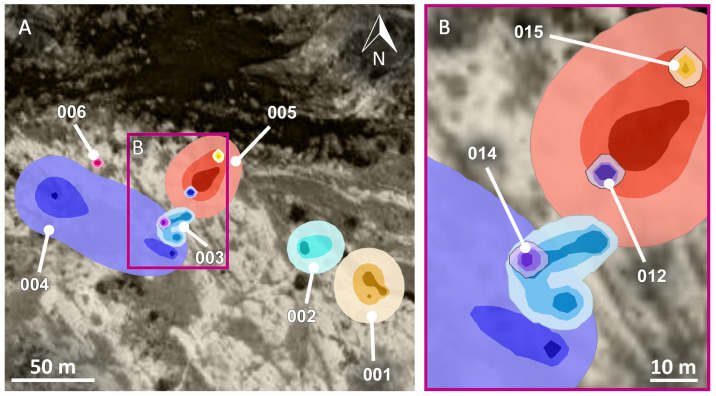
(**A**) Estimated home ranges of nine individuals of *Oplurus cyclurus* in Malaso, Isalo National Park (Madagascar) for which dBBMMs could be computed; (**B**) Individuals exhibiting overlapping home ranges.

Six individuals had overlapping home ranges, resulting in 12 cases of overlapped home ranges. In four cases, three lizards had the entire home range included in the 99% isopleth of other individuals, with two individuals even sharing (at different times) the same burrow. In the remaining cases (*n* = 8), home ranges overlapped by an average of 12% (SD = 21; range = 0.9–63.3) (Figure 2). Lastly, we failed to estimate the home range size of one individual (i.e., 011) due to excessive time gaps between observations. However, the 100% Minimum Convex Polygon for this individual was 98.5 m^2^, while KDE returned 95% fixed kernels of 602.8 m^2^ (75% KDE = 315.3 m^2^; 99% KDE = 851.1 m^2^).

### 3.2. Activity Status

**Figure 3:**
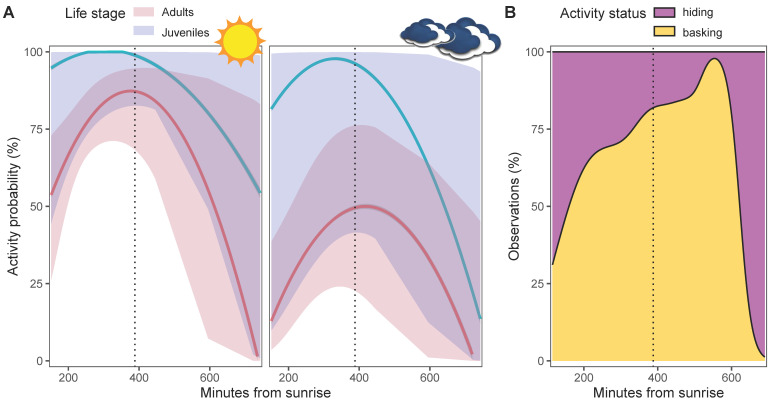
Activity pattern of 19 tracked individuals of *Oplurus cyclurus* in Malaso, Isalo National Park (Madagascar). (**A**) Predicted probability of *Oplurus* lizard activity in relation to the minutes from the sunrise during sunny days (**left**) and cloudy days (**right**); (**B**) Frequency distribution of observations of active and non-active (see legend) *Oplurus* lizards in relation to the minutes from the sunrise. In all graphs, the dotted vertical line indicates the solar noon.

The best model explaining variation in activity status had no competing models (i.e., models with ΔAIC_c_ < 2; Table S2), and indicated that the activity pattern followed a quadratic trend (binomial GLMM, LRT; χ^2^ = 9.2, df = 2, *p* = 0.009) with a peak of activity during solar noon (Figure 3A,B). Furthermore, weather conditions significantly predicted the activity of individuals, which were more active with higher temperatures (χ^2^ = 5.9, df = 1, *p* = 0.015) and less active during cloudy days (χ^2^ = 12.1, df = 2, *p* = 0.002) (Table S2). Lastly, juveniles showed a wider activity window than adults (χ^2^ = 7.4, df = 1, *p* = 0.006; Figure 3A). Past 17:00, all individuals were sheltering or in burrows (Figure 3B). No other *Oplurus* were observed past that time, even if there was still relative sunlight.

### 3.3. Burrow Use

**Figure 4:**
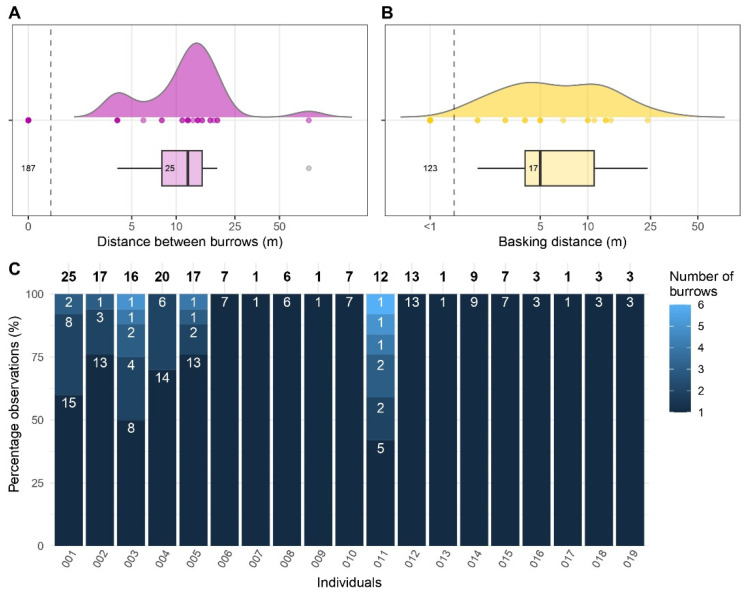
Burrow use and basking distances of 19 tracked individuals of *Oplurus cyclurus* in Malaso, Isalo National Park (Madagascar). (**A**) Frequency distribution of distances between consecutive burrows, and (**B**) frequency distribution of basking distances from the last frequented burrow. Box and density plots are plotted excluding non-movements and movements up to one meter, respectively. The number of observations used to create the boxplot is indicated within the boxplot; (**C**) Observations of burrow use in the tracked *Oplurus* lizards. Numbers on the top of the bars represent the total number of days of observation in a burrow, while numbers inside the bars refer to the number of days in which the individual was observed in the same burrow.

*Oplurus cyclurus* individuals were commonly observed seeking refuge in narrow rocky spaces, specifically utilizing crevices and natural burrows in rock formations. Burrows were on average 30.4 cm wide (range = 19.6–58.6 cm), 154.7 cm deep (range = 16.5–280 cm) and, excluding the out-and-back movements to the same burrow, the mean distance between consecutive burrows was 13.6 m (mean = 14.5; SD = 16.9; range = 4.1–79.2) (Figure 4A). Burrow features were not related to any of the morphological traits considered (i.e., total and tail lengths; Table S2).

The number of burrows used per individual ranged from one to six (Figure 4C; Table S1) and was positively correlated with the number of days of the tracking period (Pearson’s correlation r = 0.7, df = 17, *p =* 0.001). Considering those individuals tracked for more than four days (*n* = 12), the average number of burrows frequented per week was 1.1 (SD = 0.6; range = 0.5–2.9). Excluding the first observations of the tracking period (*n* = 17) and the observations of individuals monitored for less than 24 h (*n* = 3), in 87.8% of cases (*n* = 186), the individuals used the same burrow of the prior observation. Six individuals changed burrows on twenty-six occasions (i.e., 12.2% of observations), nine times of which they revisited a previously occupied burrow. The number of revisited burrows represented, on average, 32% of the total burrows frequented by these individuals (SD = 30; range = 0–67). However, considering the differences in the duration of the tracking period between individuals, this probably represents an underestimate of the actual burrow-site fidelity, as longer tracking periods resulted in more revisited burrows (Pearson’s correlation r = 0.7, df = 17, *p =* 0.001).

*Oplurus* lizards spent on average 5.9 consecutive days in the same burrow (SD = 3.9; range = 1–14), corresponding to 83.1% of the tracking period (SD = 26.6; range = 29.4–100). In cumulative terms, the amount of time (i.e., number of days) spent in a burrow by a single individual was on average 88.1% of the tracking period (SD = 19.4; range = 42–100), corresponding to 6.8 days (SD = 4.8; range = 1–15) (Figure 4C).

### 3.4. Basking Behaviour

We recorded 140 observations of basking lizards. The median distance between the basking spot and the respective burrow was 0.2 m (SD = 5.9 m; range = 0.0–24.5 m; Table S1). However, excluding the basking distances of over one meter, the median value was 4.7 m, and only five times (3.5% of observations) were individuals recorded at more than ten meters from their reference burrow (Figure 4B). Furthermore, the maximum distance of basking was not correlated with the size of the 99% dBBMM isopleth (Pearson’s correlation r = −0.1, df = 7, *p =* 0.917). Lastly, the best LMM had no competing models (Table S2) and indicated a linear increase in the basking distance with the time of the day (*t* = 2.6, *p =* 0.010).

## 4. Discussion

Overall, *Oplurus* lizards showed small home range sizes, not related to body size, and high burrow-site fidelity, with individuals occupying the same burrow for up to two consecutive weeks. Nonetheless, individuals made use of multiple burrows (up to six in our study), and even shared burrows with other conspecifics at different times. The activity pattern was unimodal, with activity and basking distance from the burrow increasing throughout the day until sunset. We argue that the saxicolous habits imposed by habitat features (the absence of trees) may result in local behavioural adjustments influencing antipredatory and foraging strategies, as well as intraspecific interactions.

### 4.1. Space Use

Lizards’ home range size is believed to result from the interplay of intrinsic (e.g., behavioural and physiological) and extrinsic factors (e.g., habitat features and resource availability) [41]. Arboreal and saxicolous lizards are predicted to have smaller home ranges than terrestrial (open ground) species [41]. The home range size of *O. cyclurus* is much smaller than the terrestrial leopard lizards *Gambelia sila* and *G. wislizenii*, in which home range can cover areas of multiple hectares [42,43], or other terrestrial iguanian lizards of comparable size (e.g., *Crotaphytus reticulatus* [44]). Conversely, our estimates largely overlap with other saxicolous (e.g., *Tropidurus hispidus* [45]) or arboreal species (e.g., *Sceloporus undulatus* [46]), including the sister species *O. cuvieri* [47] where males’ home ranges are on average 1315 m^2^ [48]. However, the possible influence of individual sex on home range size [41] was not assessed in our study, leaving the potential of a skewed estimated average home range size open.

Lizard body size is positively related to home range size and likely reflects the energetic requirements of the species [41]. *Oplurus cuvieri* is bigger than *O. cyclurus* [49], which could explain the larger home range size (approx. 30% of difference) (Table S1). Alternatively, the different methods used in other spatial ecology studies (i.e., home range size estimators, radio transmitter models, and attachment techniques) may have hampered a direct comparison of results. In the past, the most widely supported method for estimating home range size and making comparisons across studies was MCP [50]. Nonetheless, MCP is found to substantially overestimate home range areas [51], contrary to dBBMMs which currently represent the best trade-off in describing space utilization in reptiles [29].

Foraging strategies also determine the home range size of lizards, with active foragers using larger areas than ambushers or herbivorous species [7]. These differences can become evident even within the same genus of lizard, such as in the case of *G. sila* and *G. wislizenii*, with the latter having a consistently larger home range as a result of the active foraging strategy [42]. *Oplurus cuvieri* and *O. cyclurus* (and oplurids in general) are sit-and-wait predators [8,9], and their foraging strategy might therefore be reflected in their similar home range sizes. Relatedly, our estimate of home range size refers to the rainy season, when dietary needs and food availability are probably higher for this species; therefore, our estimates of home range size likely reflect the maximum across seasons.

Lastly, attachment techniques and radio transmitter mass can sometimes affect the locomotor performances of lizards [52]. The low ratio between body weight and radio-transmitter weight in our study should be a positive factor, but we cannot exclude that the kind of external affixation and external antenna, while not being as invasive as implantation, could alter the animals’ displacements, influence predation episodes, or reduce hiding capacity. However, our fieldwork showed that the technique of external tagging was effective, as we did not record any lost or dead individual nor any predation event.

### 4.2. Activity Pattern and Burrow Use

The lizards showed a unimodal activity pattern, which is common in species with high thermal tolerance, like *O. cyclurus* [15]. However, our study period was during a favourable season for the species, and it is not uncommon for activity patterns to shift to bimodality in hotter periods of the year [53].

The higher activity rates in younger individuals can be explained by differences in heating rates between age classes (smaller individuals have higher surface-to-volume ratios; e.g., [52,54]). Conversely, the basking strategy was similar between juveniles and adults, which mostly basked near their burrow entrance. In lizards, distance to the refuge is often adjusted as a trade-off between perceived predatory risk and body temperature and locomotor performance [52,55]. The tracked *Oplurus* increased the basking distance throughout the day, likely as a result of increased body temperatures and lower perceived predation risk. Habitat structural complexity and seasonality may affect predation risk in lizards [56,57]. Our study area (a rocky soil in a fairly treeless savannah) has a structurally simple habitat in which predation efficiency is predicted to be high; therefore, if the *O. cyclurus* were overexposed to predators at our site, they might have adopted a basking strategy aimed at reducing predation risk.

Our results show high burrow-site fidelity in *O. cyclurus*; however, individuals also changed burrows frequently, using alternative refuges for up to 32% of the tracking period (see Results; Figure 4C). This behaviour suggests they did not rely exclusively on specific burrows. On the one hand, in sit-and-wait forager lizards, frequent burrow change may increase feeding opportunities, and reduce predation risk and parasite load [58]. On the other hand, habitat features and shelter availability may also play a key role in determining spatial utilization and burrow use in lizards, which may also reflect on population structure [59]. For instance, the arboreal *O. cuvieri* principally makes use of a single shelter (a tree hollow [48]) and attains densities of 3.5 lizards/ha, which is nearly three times lower than our study population (i.e., 9.7 lizards/ha). Saxicolous habitats may harbour a higher number of potential shelters, which could result in different selective pressures on *Oplurus* populations. These may include alteration in territoriality, antipredatory, and foraging strategies, but also have repercussions on parasite load or persistence, ultimately reflecting on local adaptive fitness responses.

## 5. Conclusions

Despite its Least Concern status, *O. cyclurus* is facing local decline attributed to significant habitat pressure [60]. Understanding the spatial ecology of this species provides crucial insights into habitat use, territoriality, and foraging strategies, enabling targeted conservation efforts. On the other hand, emphasizing behavioural and ecological aspects of species under unconventional environmental conditions may prove valuable in supporting theory about local adaptation.

## Data Availability

Any computer codes used to generate results reported in the manuscript, as well as raw data that support the findings of this study, are available on request from the corresponding author, without undue reservation.

## Supplementary Materials

The following supporting information can be downloaded at: https://www.mdpi.com/article/10.3390/ani13203198/s1, Table S1: Table of radio-tracked individuals of *Oplurus cyclurus* in Isalo Massif (central-southern Madagascar); Table S2: List of Generalized and Linear Mixed models explaining the activity status, the basking behaviour and the burrow use of *Oplurus cyclurus* in Isalo Massif (central-southern Madagascar).
