# Supplementary material for: Spatial Ecology of an Arboreal Iguana (Oplurus cyclurus) in a Treeless Landscape
Source: Animals (Basel). 2023 Oct 13;13(20):3198. doi: 10.3390/ani13203198 (PMC10603757; doi:10.3390/ani13203198)
Supplement: Supplementary file 1 [file animals-13-03198-s001.zip › animals-2512424-supplementary.pdf]

animals

Article

Spatial Ecology of an Arboreal Iguana (*Oplurus cyclurus*) in a Treeless Landscape

Fulvio Licata, Paolo Eusebio Bergò, Devin Edmonds, Franco Andreone and Gonçalo M. Rosa

Supplementary Materials

Table S1. Radio-tracked individuals of *Oplurus cyclurus* in Isalo Massif (central-southern Madagascar).

Table S2. List of Generalized and Linear Mixed models explaining the activity status, the basking behaviour and the burrow use of *Oplurus cyclurus* in Isalo Massif (central-southern Madagascar).

Copyright: © 2023 by the authors. Submitted for possible open access publication under the terms and conditions of the Creative Commons Attribution (CC BY) license (https://creativecommons.org/licenses/by/4.0/).

Table S1. Radio-tracked individuals of *Oplurus cyclurus* in Isalo Massif (central-southern Madagascar). The asterisk indicates the home range estimated for individual #011 which has been calculated using Kernel Density Estimator, while between parenthesis is reported the 100% Minimum Convex Polygon. TL and TaL indicate the total length and tail length of the individual (in mm), respectively. Life stages: A and J refer to adult and juvenile, respectively.

ID	Tracking period (days)	*n* observations	Life stage	TL	TaL	Time between observations (hours)	*n* burrows used	*n* burrow changes	*n* re-used burrows	Distance between burrows (m)	Basking distance (m)	Isopleth 75% (m²)	Isopleth 95% (m²)	Isopleth 99% (m²)
001	31	46	A	-	-	16.2 ± 17.9 (0.8–75.5)	3	5	2	12.18 ± 0.31 (12.04–12.73)	0.20 ± 0.71 (0.05–4.1)	76.35	391.06	1520.74
002	23	25	A	162	91	22.3 ± 15.7 (1–73.9)	3	5	2	7.99 ± 5.30 (4.12–14)	2.39 ± 6.76 (0.05–24.5)	32.99	227.87	862.85
003	22	25	A	134	64	21.3 ± 20.2 (0.9–96.6)	5	4	–	9.70 ± 4.61 (6–14.87)	2.81 ± 4.98 (0.05–13.8)	74.39	201.65	373.45
004	22	26	A	152.8	72.8	20.4 ± 16.6 (0.9–75.7)	2	1	–	79.16	0.52 ± 0.80 (0.05–3.14)	15.28	762.13	4650.71
005	21	22	A	145	59	23.3 ± 16.7 (0.4–74.1)	4	4	1	17.70 ± 2.78 (13.60–19.42)	2.05 ± 4.58 (0.05–13.25)	145.89	585.82	1764.41
006	8	13	A	165.5	76.3	13.9 ± 14.5 (1–44.9)	1	–	–	–	0.45 ± 0.80 (0.05–2.5)	6.65	14.95	35.96
007	4	2	J	76.2	43.5	72	1	–	–	–	5.05 ± 6.72 (0.3–9.8)	–	–	–
008	7	6	A	-	-	28.4 ± 50.9 (0.6–118.3)	1	–	–	–	0.47 ± 0.60 (0.05–1.15)	–	–	–
009	2	2	J	80	43.65	23.90	1	–	–	–	0.05	–	–	–
010	8	5	A	-	-	41.6 ± 53.3 (0.5–120)	1	–	–	–	0.06 ± 0.03 (0.05–0.1)	–	–	–
011	15	14	A	172.1	96.9	26.3 ± 18.8 (0.0–74.7)	6	7	2	11.90 ± 4.71 (4.47–17.03)	1.80 ± 1.98 (0.05–4.7)	315.26*	602.79*	851.12 (98.50)*
012	14	15	J	69.8	21	22.9 ± 18.3 (0.8–74.2)	1	–	–	–	1.92 ± 3.26 (0.05–9.7)	7.40	17.89	38.87
013	1	2	A	-	-	0.70	1	–	–	–	0.30 ± 0.35 (0.05–0.55)	–	–	–
014	10	10	A	173	89	24.8 ± 5.5 (14.9–33.0)	1	–	–	–	0.12 ± 0.08 (0.05–0.2)	5.28	17.59	43.27
015	8	8	A	143.5	79	25.3 ± 6.3 (15.3–32.8)	1	–	–	–	0.39 ± 0.51 (0.05–1.12)	3.98	11.71	39.45
016	4	4	A	-	-	26.9 ± 4.8 (23.3–32.3)	1	–	–	–	0.40 ± 0.14 (0.3–0.5)	–	–	–
017	1	1	J	86	45	–	1	–	–	–	0.05	–	–	–
018	4	4	A	-	-	26.5 ± 4.9 (23.4–32.1)	1	–	–	–	0.45 ± 0.57 (0.05–0.85)	–	–	–
019	4	4	A	-	-	26.4 ± 5 (23.1–32.2)	1	–	–	–	0.36	–	–	–

Table S2. List of Generalized and Linear Mixed models explaining the activity status, the basking behaviour and the burrow use of *Oplurus cyclurus* in Isalo Massif (central-southern Madagascar). The table shows the number of parameters estimated in the model (K), AIC_c score of the model, AIC_c difference with the best model (ΔAIC_c), the relative weight of the model with respect to the candidate set of models (AIC_c *wt*), variation explained by the fixed effects (R^2_M = marginal R^2) or by both fixed and random effects of the model (R^2_C = conditional R^2). Models which had a simpler nested model with lower AIC_c were removed. In the best models (underlined), significant variables are highlighted in bold. Time = minutes from sunrise; Stage = ontogenetic stage (adult vs. juvenile); Cloud coverage = cloud coverage (no clouds, partially cloudy, cloudy); Temperature = temperature at 1.2 m above the ground.

**Response**<br>**Independent variables**	**K**	**AIC_c**	**ΔAIC_c**	**AIC_c *wt***	**R^2_M**	**R^2_C**
**Activity status**						
<u>**Time + Time^2 + Stage + Cloud coverage + Temperature**</u>	8	145.82	0.00	0.73	0.55	0.56
Time + Time^2+ Stage + Cloud coverage	7	149.48	3.67	0.12	0.55	0.55
Stage + Cloud coverage + Temperature	6	150.58	4.76	0.07	0.41	0.43
Time + Time^2 + Cloud coverage + Temperature	7	150.99	5.17	0.05	0.41	0.46
Time + Time^2 + Stage + Temperature	6	153.51	7.70	0.02	0.52	0.52
Cloud coverage + Temperature	5	154.70	8.88	0.01	0.32	0.38
[…]						
Null model	2	187.40	37.92	<0.001	–	–
**Basking distance**						
<u>**Time + Time^2**</u>	5	279.75	0.00	0.98	0.08	0.09
Null model	3	287.99	8.24	0.02	–	–
**Burrow choice (depth burrow)**						
Null model	3	46.50	–	–	–	–
**Burrow choice (width burrow)**						
Null model	3	14.80	–	–	–	–

Disclaimer/Publisher's Note: The statements, opinions and data contained in all publications are solely those of the individual author(s) and contributor(s) and not of MDPI and/or the editor(s). MDPI and/or the editor(s) disclaim responsibility for any injury to people or property resulting from any ideas, methods, instructions or products referred to in the content.
